# Enhanced Photocatalytic Activity of Zn-Al Layered Double Hydroxides for Methyl Violet and Peat Water Photooxidation

**DOI:** 10.3390/nano12101650

**Published:** 2022-05-12

**Authors:** Is Fatimah, Amri Yahya, Rendy Muhamad Iqbal, Muchammad Tamyiz, Ruey-an Doong, Suresh Sagadevan, Won-Chun Oh

**Affiliations:** 1 Department of Chemistry, Faculty of Mathematics and Natural Sciences, Universitas Islam Indonesia, Kampus Terpadu UII, Jl. Kaliurang Km 14, Sleman, Yogyakarta 55584, Indonesia; amri.yahya@students.uii.ac.id; 2 Department of Chemistry, Faculty of Mathematics and Natural Sciences, Universitas Palangka Raya, Palangka Raya 27111, Indonesia; iqbalrm@mipa.upr.ac.id; 3 Universitas Nahdlatul Ulama Sidoarjo, Jl. Monginsidi Kav DPR No. Dalam, Sidoklumpuk, Sidokumpul, Sidoarjo 61218, Indonesia; m_tamyiz.tkl@unusida.ac.id; 4 Institute of Analytical and Environmental Sciences, National Tsing Hua University, Hsinchu 300044, Taiwan; radoong@mx.nthu.edu.tw; 5 Nanotechnology & Catalysis Research Centre, University of Malaya, Kuala Lumpur 50603, Malaysia; drsureshnano@gmail.com; 6 Department of Advanced Materials Science and Engineering, Hanseo University, Seosan-si 356-706, Korea

**Keywords:** dye degradation, layered double hydroxides, photocatalyst, peat water, photooxidation

## Abstract

Zn-Al Layered Double Hydroxides (Zn-Al LDHs) and its calcined form were successfully prepared and utilized for the removal of methyl violet (MV) and treatment of peat water by photocatalytic oxidation. The research was aimed to evaluate the effect of calcination to Zn-Al LDHs for the effect on the physicochemical character and the capability as a photocatalyst. The characterization of the samples was investigated by X-ray diffraction (XRD), scanning electron microscopy (SEM), transmission electron microscopy (TEM), Brunauer–Emmet–Teller specific surface area (BET), and X-ray photoelectron spectroscopy (XPS). The results showed that the increased BET specific surface area along with the enhanced porous structure was achieved by the calcination procedure, which is associated with the enhanced interlayer space of d_003_ identified by XRD analysis. Thermal conversion showed an influence to the increased band gap energy from 3.10 eV in the uncalcined Zn-Al LDHs into 3.16 eV for the calcined material. These character changes contributed to the enhanced photocatalytic activity of the Zn-AL LDHs by calcination, which was proposed and verified by experiments. It was observed that photocatalytic activity of the material for MV gave about a 45.57% removal of MV and a 68% removal for the natural organic material of the peat water.

## 1. Introduction

Photocatalytic oxidation has been recognized as potential method for organic compounds-contaminated water treatments, including wastewater containing dyes. It is estimated that more than 10,000 kinds of dye were utilized in many industries that contribute the wastewater’s high polluted condition [1]. The loss of dye use in such industrial processing gives characteristics of wastewater such as a bad odor, not being able to properly release in an aquatic environment, high toxicity, and persistence. Therefore, effective treatment and serious handling are required, and this is done to support small industries utilizing colorants such as batik industries. Another organic containing water is peat water, which is a problem in some places in southeast Asia including Malaysia and Indonesia, and it is greater than 24.8 Mha of the area [2]. Peat water exists due to the peatlands of tropical peat swamp forests that are most extensive in Southeast Asia, including in the islands of Borneo and Sumatra and on Peninsular Malaysia. Peatlands are composed from the accumulation of sediment formed by the residues of weathered past plant tissue or fossilized plant matters, with a thickness of more than 50 cm. By the lack of oxygen, the leached components intrusions form the peat water with the characteristic of containing a heterogeneous mixture of organic compounds with various chemical functional groups, structures, and molecular weights. The peat water characteristics such as high chemical oxygen demand (COD), natural organic matter (NOM), a black color, and high total suspended solids (TSS) are unacceptable to be directly consumed, but its availability follows the soil and area conditions [2,3]. The exploration for photocatalytic treatment with efficiencies and reusability of the process is a great challenge to provide a low-cost photocatalytic treatment. The explorations within this scheme consist in the preparation of recyclable photocatalysts [4].

Among some metal oxide semiconductors, zinc oxide is a remarkable metal oxide semiconductor with high photocatalytic performance and a low production cost. However, the practical applications of ZnO sometimes faced limited conditions such as a rapid loss of activity. The photoactive Zn-Al layered double hydroxide (LDHs) is one of the explored materials that is an effective photocatalyst for some organic pollutant’s degradation. LDHs are a class of synthetic clays with brucite [Mg(OH)_2_]-like cationic layers containing anions in the hydrated interlayer for the charge balance. Nonetheless, LDHs have layered structures that include them into clay structured materials and that have the capability to adsorb anionic compounds, involving dye and ionic organic compounds by the ion exchange mechanism. This role has a potentially enhanced effectivity in the photocatalytic mechanism. Zn-Al LDHs were reported to have high effectivity in the degradation of methylene blue and rhodamine B and some other organic compounds [5,6,7]. Based on the contribution of the ionic adsorption mechanism in the photocatalytic process, it can be hypothesized that Zn-Al LDHs have the capability to be utilized in peat water treatment; in addition, the use for peat water treatment was not reported yet.

Due to the role of surface interaction among the target molecule and the photocatalyst for a whole degradation mechanism, the surface character of Zn-Al LDHs determines the capability of the photocatalyst. The surface modifications to the composite were strategies to form a larger surface capacity. As an example, the modification of Zn-Al LDHs with methylimidazole and the sodium format created the lamellar structure to form a three-dimensional structure with a higher specific surface area [8]. Similarly, the surface interaction of CO_2_ as a molecule target can be governed by Cu^2+^ modification to Zn-Al LDHs with enhanced photocatalytic activity [8]. The exfoliated structure was achieved by the combination of surfactant modification with calcination to provide a larger area that elevated photocatalytic capability [9,10,11]. By a simpler method, calcination importantly influenced the removal of salicylic acid by Zn-Al LDHs; specifically, the optimum calcination temperature for creating higher specific surface area is in line with the photocatalytic degradation capability [12].

Considering the potency of Zn-Al LDHs in industrial and applied scales, the reusability and the stability of the photocatalyst are the important features. As the calcination preserved a higher surface area, this research was aimed to evaluate the use of Zn-Al LDHs and the effect of calcination to its photocatalytic activity and stability. Although there are many previous works on the photodegradation activity of Zn-Al LDHs, the photocatalytic efficiency as a function of calcination treatment was not addressed. In addition, the photocatalytic activity of Zn-Al LDHs for peat water treatment was not reported yet; meanwhile, it is an important issue to be resolved for a sustainable development of some areas in the Southeast Asia region.

The instrumental analysis towards the structure of Zn-Al LDHs was performed by using X-ray diffraction (XRD), scanning electron microscopy–energy dispersive X-ray (SEM-EDX), transmission electron microscopy (TEM), and UV–visible diffuse-reflectance spectroscopy (UV-DRS); meanwhile, the stability of photocatalyst was evaluated by means of X-ray photoelectron spectroscopy (XPS) analysis. Due to the vast use in many dying industries, methylene violet (MV) was used as dye model, and peat water from Palangka Raya region was selected to be treated.

## 2. Materials and Methods

### 2.1. Materials

Chemicals consisting of zinc acetate (MW: 219.5 g/mol; 99%), aluminum chloride (MW: 133.3 g/mol, >98%), methyl violet (MV) (MW: 407.9 g/mol, >88%), ethylene diamine tetra acetate (EDTA) (MW: 336.2 g/mol, >98.5%), isopropanol (IPR) (MW: 60.1 g/mol, >99.5%), and H_2_O_2_ (MW: 34.0 g/mol, 30%) were purchased from Merck (Darmstadth, Germany) and were of analytical grade and utilized without any purification. Peat water was sampled from Universitas Palangka Raya Park.

### 2.2. Synthesis of Zn-Al LDHs

The Zn-Al LDHs was prepared by the hydrothermal method. The solutions of zinc acetate and aluminum chloride in the molar ratio of 3:1 were mixed under stirring for an hour before the NaOH solution was added to obtain a pH of 10 ± 0.5 with the speed of the addition of 4 mL/min. The mixing produced a gel, which was mixed and inserted in a Teflon-lined autoclave for the hydrothermal method at a temperature of 150 °C overnight. The gel from the treatment was dried in an oven at 60 °C followed by calcination at 400 °C for 4 h. The selection of a temperature of 400 °C was based on the optimum temperature giving the increasing specific surface area from a previous work [12], and it was based on the stable condition of thermal change on the differential thermal analysis–thermogravimetric analysis (DTA-TGA). The crushing to the solid obtained from these steps gave the Zn-Al LDHs powder.

### 2.3. Characterization of Zn-Al LDHs

The Zn/Al-LDHs powder was characterized with X-ray diffraction (XRD) on a Rigaku D/max-2550 V diffractometer (Tokyo, Japan). The Ni-filtered Cu K radiation (χ = 1.540 56 Å) was utilized as radiation source with an analytical condition of the 2-theta range of 10–80° and the scanning rate: 0.02° s^−1^. The thermal property of Zn-AL LDHs was studied by differential thermal analysis–thermogravimetric analysis (DTA-TGA) on a Seiko instrument. The surface morphology of the sample was examined with field-emission scanning electron microscopy (FESEM, JEOL JSM-6700F, Tokyo, Japan) equipped with energy dispersive X-ray spectrophotometry (EDS), and transmission electron microscopy (TEM, JEOL JEM-200CX, Tokyo, Japan, working at 160 kV) was employed for more-detailed analysis. The optical properties of nanoparticles were studied by using diffuse reflectance UV–visible spectroscopy (UV-DRS JASCO V760, Tokyo, Japan). X-ray photoelectron spectroscopy (XPS) analysis was performed on a V.G. Scientific ESKALAB MKII (Tokyo, Japan) instrument utilizing a monochromatic Al *K*_α_ radiation with a photon energy of 1486.6 ± 0.2 eV. 

### 2.4. Photocatalytic Activity and Adsorption Study of Zn-Al LDHs

Photocatalytic activity of Zn-Al LDH was assessed on the photocatalytic oxidation (photooxidation) of MV as a pollutant model and also to the peat water. In particular, for each photocatalytic test, about 1 g of the powder dispersed in 1000 mL of the MV solution (20 mg/L) with the addition of 1% H_2_O_2_ and was kept under a dark situation around 30 min for gaining adsorption–desorption equilibrium before illumination. For each experiment after adsorption equilibrium, the mixture was exposed under a UV lamp (296 nm, Philips, 40 W), and then the sampling was performed at every 15 min. The changes in MV concentration were monitored by using UV–visible spectrophotometry (HITACHI U-2010, Tokyo, Japan) in the range of 400–800 nm in wavelength. The photodegradation efficiency (DE) was calculated using the following Equation (1):

(1) $$DE\left(\%\right)=\left(1-\frac{C_{t}}{C_{0}}\right)\;\times \;100$$

where *C_t_* and *C*_0_ are the concentrations of MV at the time of sampling *t* and at the initial condition, determined from the colorimetric method at the wavelength of 589 nm. To ensure the degradation to the MV, HPLC analysis was also performed.

The photocatalytic activity of the sample to reduce organic components in peat water was evaluated based on natural organic matter (NOM) analysis by means of specific ultraviolet absorbance (SUVA) at 254 nm on NOM and the chemical oxygen demand (COD) reduction [3,13]. The COD was measured by chromate oxidation followed by spectrophotometric analysis. To examine the photocatalytic mechanism, a study on the adsorption capability of the sample was also conducted with same condition but without the light illumination.

## 3. Results

### 3.1. Materials Characterization

**Figure 1:**
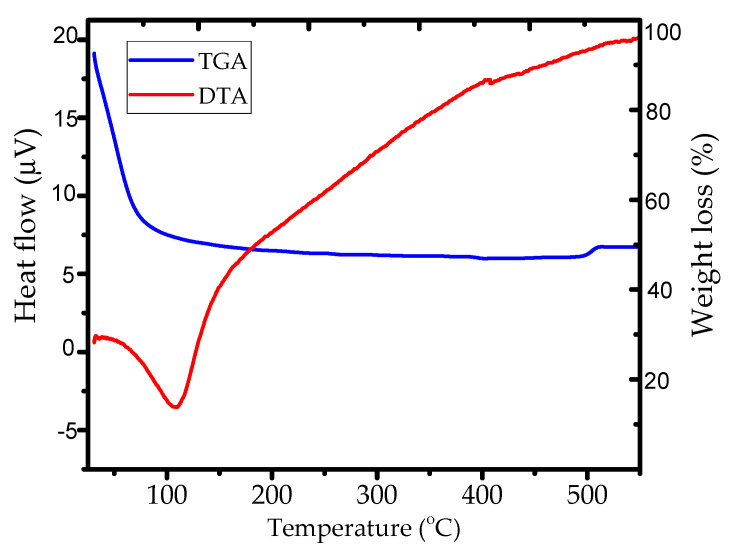
Thermogram of Zn-Al LDHs.

The study on the synthesis and identification of Zn-Al LDHs material is mainly on the effect of calcination on the structure and some properties such as crystallinity, the specific surface area, and the optical properties of the material. A calcination temperature of 400 °C was selected based on previous work that identified the effect of temperature on the adsorption performance of Zn-Al LDHs [12]. In addition, the confirmation was obtained based on the DTA-TGA thermogram as presented in Figure 1.

The plots from the DTA-TGA analysis show that the important peak corresponding to the weight loss and structural change was at around 100–125 °C. This is referred to as the thermal change related with dehydration or the loss of water from LDHs structure. With the advanced increasing temperature, there was no significant change in weight loss, but the thermal effect showed an endothermic effect. Regarding the pattern of the plots, it can be concluded that the mass was stable at around 200–480 °C, and there was a stabilized endothermic effect after 400 °C.

**Figure 2:**
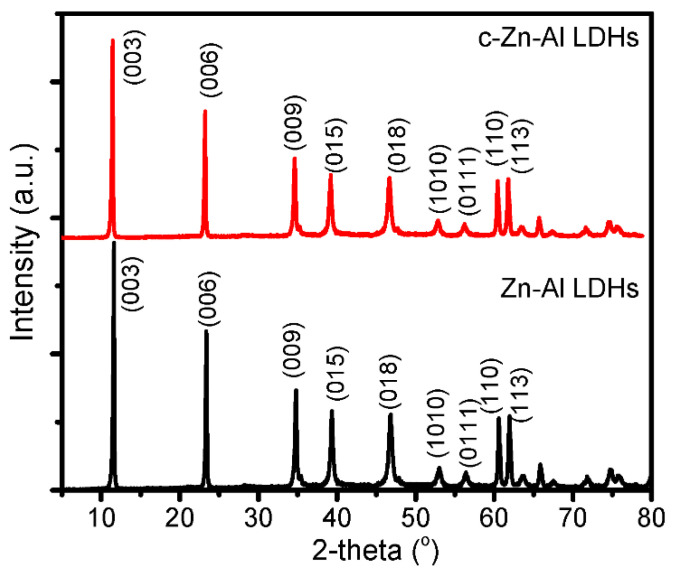
XRD patterns of Zn-Al LDHs and c-Zn-Al LDHs.

The structure identification of Zn-Al LDHs and the calcined form (c-Zn-Al LDHs) was observed by XRD analysis, and the results are shown in Figure 2.

Both samples show the sharp diffraction peaks associated with (003), (006), (009), (015), (018), (010), (1010), (0111), (110), and (113) diffraction planes of the LDHs structure, which was following the database of JCPDS number 38-0486 [7,14]. It was observed that a similar pattern appeared for c-Zn-Al LDHs, which confirmed the formation of the hydrotalcite lamellar structure. From the d_003_ values of both samples, a smaller angle of the reflection in c-Zn-Al LDHs is associated with the higher interlayer space caused by the calcination process. The d_003_ of Zn-Al LDHs and c-Zn-Al LDHs are associated with the values of 7.93 and 8.09 nm, respectively, indicating the opening space that aimed to obtain better adsorption and photocatalytic capability. This phenomenon was similar with what was reported by calcining Zn-Al LDHs, calcined Mg-Al LDHs, and Zn-Al LDHs intercalated with Ag [15,16,17]. By using the (003), (006), and (009) peaks, the crystallite size of LDHs were calculated based on the Scherer equation (Equation (2)):

𝑑 = 𝑘𝜆/(𝐵cos𝜃) (2)

where 𝑑 is the mean crystalline size of LDHs; 𝜆 is the wavelength of radiation (1.5406 Å); 𝜃 is the angle of the selected reflection; and 𝐵 is the intensity of the full width at half maximum (FWHM) of the selected reflection. The parameters are presented in Table 1.

From the calculations, it can be concluded that the mean crystallite size of LDHs reduced over the thermal treatment, from 81.4 nm into 64.4 nm.

**Figure 3:**
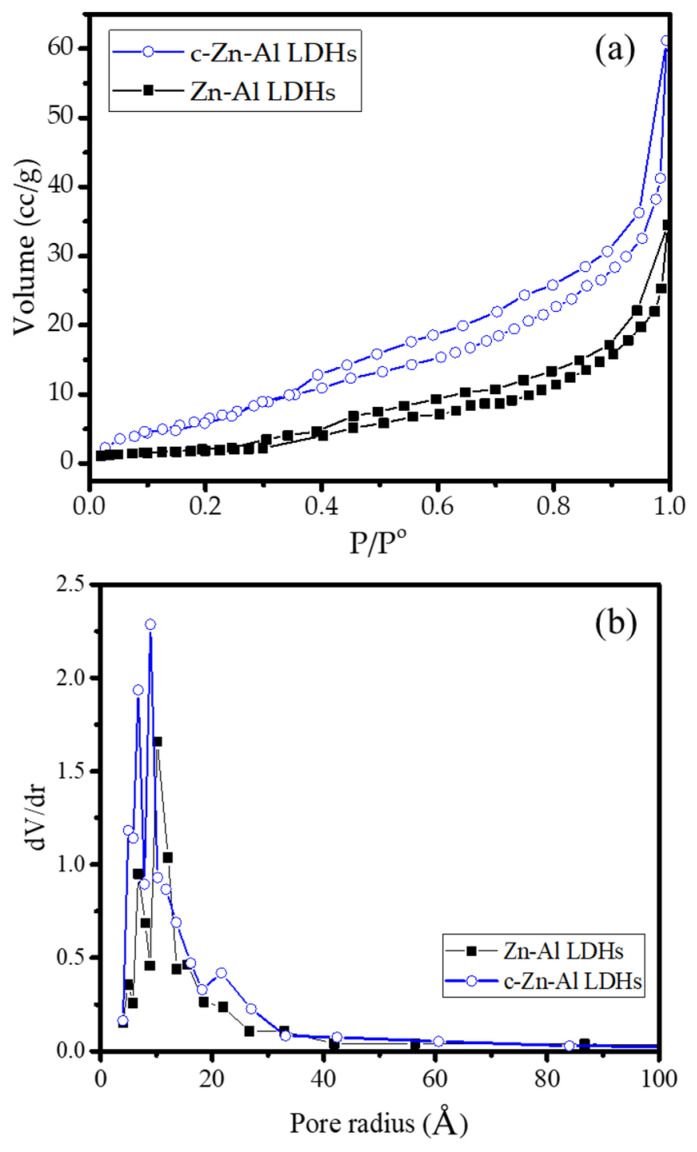
(**a**) Adsorption–desorption, and (**b**) pore distribution of materials.

The increasing d_003_ space and also the change in crystallite size that indicated the increasing distance of the layers was also identified by the surface profile from the adsorption–desorption isotherm and the pore distribution presented in Figure 3 and the calculated parameters presented in Table 2.

The increasing adsorption capability was identified throughout the P/P^0^ range, which was also reflected by the increasing volume from the pore distribution and the calculated parameters presented in Table 2.

**Figure 4:**
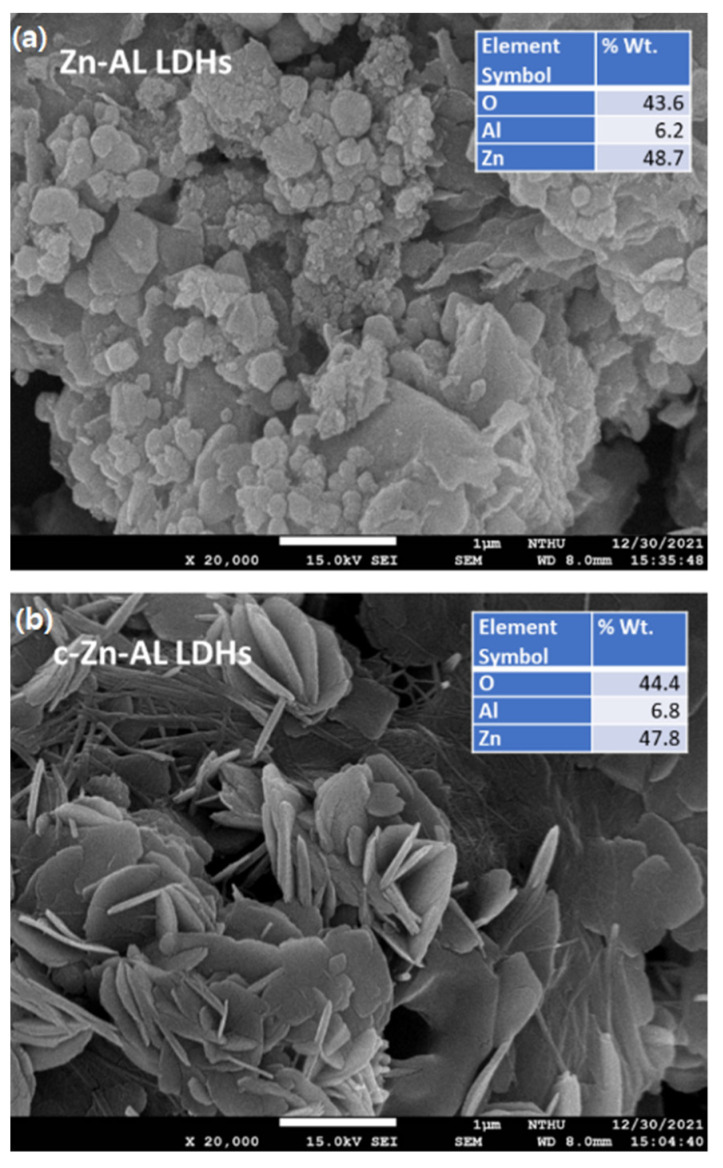
SEM-EDS of Zn-Al LDHs (**a**) and c-Zn-Al LDHs (**b**).

**Figure 5:**
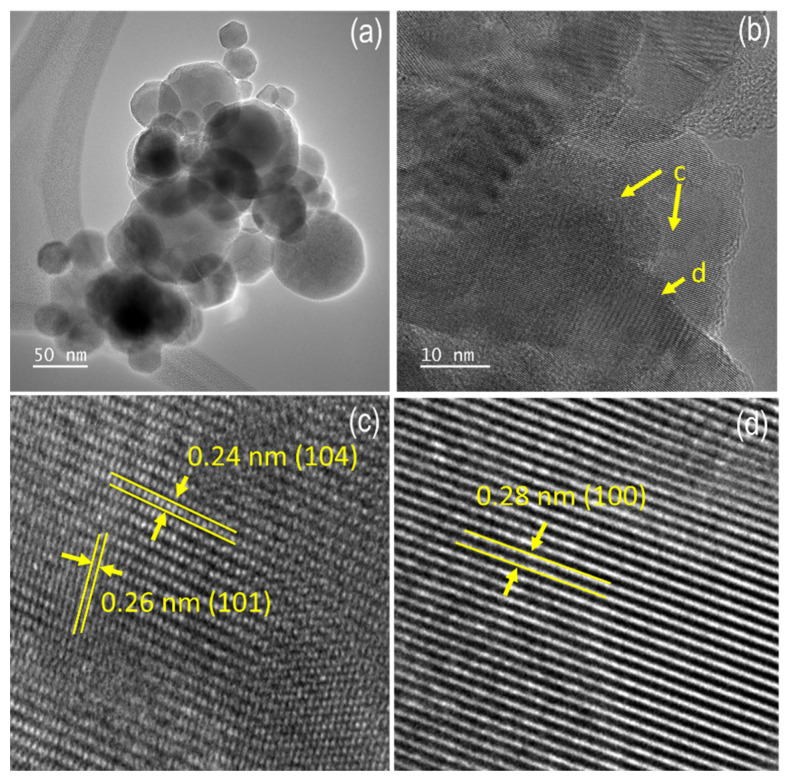
(**a**,**b**) TEM image of c-Zn-Al LDHs in different magnification; (**c**) The identified lattice fringes associated with LDHs structure; (**d**) The identified lattice fringes associated with ZnO.

The enhancements in surface parameters consisting of the BET specific surface area, the pore volume, and the pore radius were, however, not reflected significantly by the SEM-EDS measurements presented in Figure 4. The SEM images suggest that there was a significant influence of the calcination and pore opening to the physical morphology of the LDHs. The EDS analysis results also imply that the calcination did not chemically affect the composition, which conclusively stated that the calcination gave the physical influence especially for the structure of the layers but not affecting the chemical composition. Furthermore, the layer structures were confirmed by the TEM analysis presented in Figure 5b. The identified lattice fringes from Figure 5c,d appeared with the interplanar distances of 0.24 and 0.26 nm, which are ascribed to the (104) and (101) plane of the hexagonal Zn-Al LDHs phases. In addition, some spots show the lattice fringes of 0.28 nm, which is associated with the (100) reflection of ZnO [16,18,19,20].

**Figure 6:**
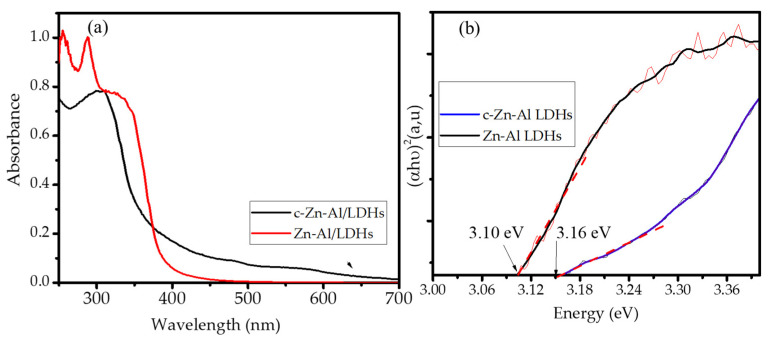
(**a**) UV-Vis DRS absorption spectra and (**b**) Tauc plots of materials.

The light absorption characteristics of the Zn-Al LDHs was studied using UV-DRS analysis with the Tauc plots presented in Figure 6. As shown in Figure 6, the shift in the absorption spectrum into the lower wavelength is shown by c-Zn-Al-LDHs along with the reduced absorbance. The shift is related with the smaller crystallite size of the material and consisted with the calculated band gap energy of 3.10 and 3.16 eV (as shown by intersection lines in red) for Zn-Al LDHs and c-Zn-Al LDHs, respectively [21]. The values were less than the band gap energy of the bulk and nano ZnO, which were around 3.2 eV [22,23]. The lower band gap energy was associated with the Zn oxidation states and the chemical environment in the LDH structure that was obviously affecting the valence band of the Zn.

### 3.2. Adsorption and Photocatalytic Studies

**Figure 7:**
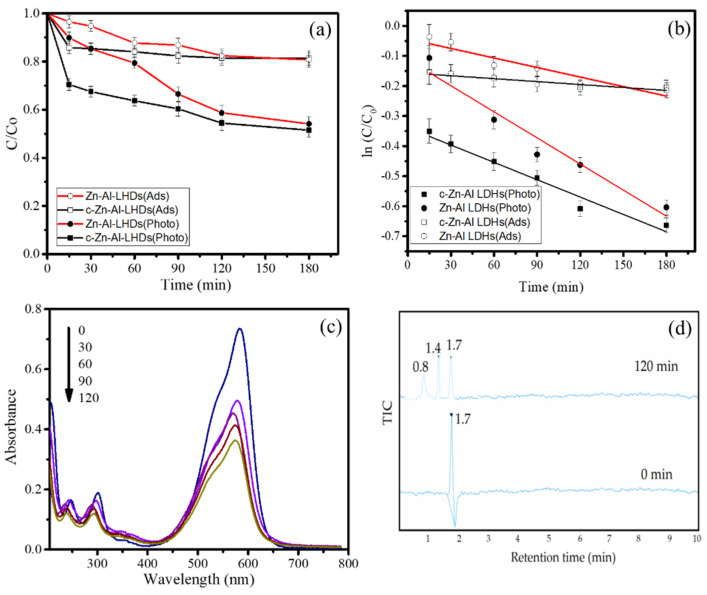
(**a**) Kinetics of adsorption and photooxidation of MV using materials. (**b**) Pseudo-first-order kinetics plot of adsorption and photooxidation of MV. (**c**) UV–visible spectra of treated solution. (**d**) HPLC results of initial and treated solutions.

To investigate the photocatalytic activity of Zn-Al LDHs and the effect of calcination, the MV photocatalytic oxidation was performed. In particular, the studies were conducted with a 20 ppm MV concentration and a catalyst dose of 1 g/L under a 296 nm UV-light and an additional H_2_O_2_ (1%). In order to evaluate the difference between the photocatalytic mechanism and the adsorption mechanism for decreasing MV concentrations, adsorption experiments were also conducted. The kinetics plot in Figure 7 exhibits that there was photocatalytic performance of both calcined and uncalcined Zn-Al LDHs compared to the adsorption kinetics, which were due to the faster degradation of MV. As c- Zn-Al LDHs adsorption gave a faster reduction in MV concentration compared to Zn-Al LDHs, the kinetics from photocatalytic oxidation were also similar. This conclusively obtained that the specific surface area plays an important role for enhancing the oxidation mechanism, and it appropriately meets with either the theoretical aspect or similar works that stated that surface interaction is significantly included in the degradation mechanism [24]. Moreover, as proof of the degradation of MV structure, the wavelength with optimum absorbance in the UV–visible spectra of MV shows the shift over the time of treatment (Figure 7b). Referring to previous studies on methyl-containing dyes degradation, this phenomenon is associated with de-methylation as the initial step for further degraded molecules [25,26]. The HPLC analysis result presented in Figure 7d confirmed this assumption. It can be seen that the treated solution for 120 min shows not only the peak corresponding to MV but also two other peaks as the results of the degradation products.

The kinetics studies to the adsorption and photooxidation revealed that both processes were studied with pseudo-first-order kinetics and pseudo-second-order kinetics—refer to the following equations (Equations (3) and (4)):

(3) $$ln\frac{C_{t}}{C_{0}}=-kt$$

(4) $$\frac{1}{C_{t}}=kt+\frac{1}{C_{0}}$$

where *C_t_* and *C*_0_ are the MV concentration at time *t* and at the initial condition, and *k*_1_ and *k*_2_ are the kinetics constants of pseudo-first order and pseudo-second order, respectively. The calculated parameters from the studies are presented in Table 3. The R^2^ data that are also reflected by the graphs in Figure 7b suggest that all the processes obeyed pseudo-first-order kinetics, meaning that the concentration of MV is a significant factor for either adsorption or the photooxidation rate.

From the kinetics plots of adsorption by both materials and the data in Table 3, it is seen that the adsorption pattern is in line with the photooxidation as the DE values over c-Zn-Al LDHs were higher compared to those by Zn-AL LDHs. By this phenomenon, it is interesting to evaluate the adsorbate–adsorbent interaction on the adsorption process. The study was performed by evaluating the adsorption isotherm using the Langmuir and Freundlich isotherms, with the following equations (Equations (5) and (6)):

(5) $$q_{e}=\frac{q_{m}K_{L}C_{e}}{1+K_{L}C_{e}}\;$$

(6) $$q_{e}=K_{F}C_{e}^{1/n}$$

where *q_e_* and *q_m_* (mg/g) are the adsorption capacities of the adsorbent at the equilibrium and maximum values, respectively; *C_e_* (mg/L) is the concentration of the adsorbate in equilibrium; *K_L_* (L/mg) is the Langmuir constant related to the energy of adsorption and adsorption intensity; and *K_F_* and *n* are the Freundlich constants related to the adsorption–desorption equilibrium [27,28]. From both isotherms, the calculated isotherm parameters are presented Table 4.

From the R^2^ parameters, it was conclusively obtained that the adsorption by both materials fit to the Freundlich isotherm model rather than the Langmuir isotherm. It was also seen by the comparison of the *K_F_* and *K_L_* values that there were significant improvements in the adsorption capability. In addition, the 1/*n* values were higher than 1, which suggests that the chemical interactions dominantly occurred [29,30]. These results implied the existing chemisorption as part of the whole process in photocatalytic degradation [29].

**Figure 8:**
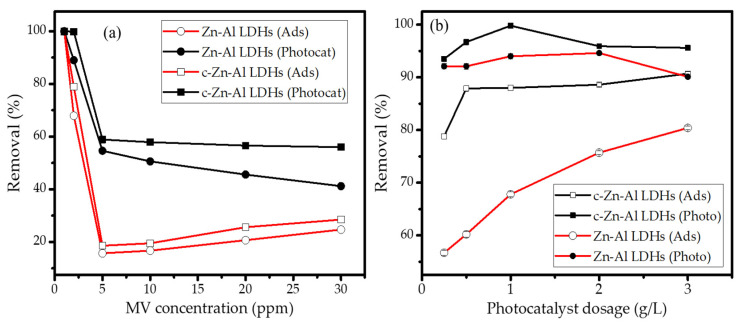
(**a**) Effect of initial concentration of MV on removal; (**b**) effect of photocatalyst dosage on removal.

In order to ensure the significant role of photooxidation besides the adsorption mechanism, the evaluation on the effect of the MV initial concentration and the photocatalyst dosage on the removal efficiency were conducted, and the data are presented in Figure 8.

The influence of MV concentration was studied at the range of 1–30 ppm at the condition of the photocatalyst dosage of 1 g/L; meanwhile, the effect of the photocatalyst dosage was studied for 10 ppm of the MV solution.

The pattern presented in Figure 8a suggests that the increasing MV concentration tends to reduce the removal efficiency of either adsorption or photooxidation by both Zn-Al LDHs and c-Zn-Al LDHs. In general, the intensive adsorption was achieved at low concentrations as the rapid removal was higher at an enormous number of available active adsorption spots on the material’s surface [31]. In more detail, the removal of around 90–100% was achieved at the initial concentration of 1–5 ppm over the photocatalysis process. By the concentration variation, it also can be seen that the appreciable enhancement by photooxidation instead of adsorption appeared, mainly at the high MV concentrations. In addition, the remarkable increasing removal was also exhibited by the calcined photocatalyst. The role of photooxidation is obviously presented by the effect of photocatalyst dosage. In more detail, the amount of photocatalyst in the reaction system influences both the adsorption and the photooxidation mechanism and has an optimum condition of around 2 g/L. The additional amount of photocatalyst tends to maintain the removal by adsorption and moreover reduces the removal over photooxidation. The reduced removal at the increasing photocatalyst amount is caused by the limited light penetration into the solution at the more turbid solution by the photocatalyst particles. A similar phenomenon was also reported from similar works on the use of a clay-based photocatalyst such as TiO_2_/pillared clay, Fe_2_O_3_/montmorillonite, and ZnO-supported clay [29,32,33].

Based on the DE value, the MV removals obtained in this work were not as high as the use of other photocatalysts such as ZnO, PbTiO_3_, TiSiW_12_O_40_/TiO_2_, or 3D MOF [34,35,36,37]. The DE values by these photocatalysts were around 80–99%; in particular, the values obtained by ZnO and TiO_2_ were 90% and 94% for 90 min of treatment [34,36]. Some factors in the photocatalytic testing such as photocatalyst dosage and MV concentration are the crucial factors that gave different effectivities. As an example, the initial MV utilized in the use of PbTiO_3_ was 5 ppm with the photocatalyst dose of 3 g/30 mL and the dosage of 0.3 g/L for 10 ppm of MV by ZnO [34,36]. In the other MV photodegradation experiment, the DE of 85% by 3D MOF at the dosage was 8 g/L for 5 ppm of MV [37]. As the photocatalyst dose utilized in this work was 1 g/L and the initial concentration of MV was 10 ppm, the DE in this work was in acceptable values (95.9%). The comparison of MV degradation by photooxidation over various photocatalysts is listed in Table 5.

**Figure 9:**
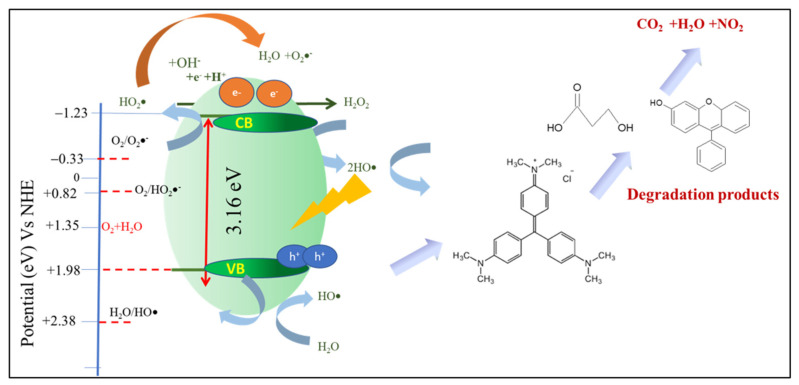
Schematic diagram of photocatalysis mechanism over Zn-Al LDHs.

Based on the valence band diagram of some LDHs and the calculated band gap energy of 3.16 eV in Zn-AL LDHs, the possible photocatalytic mechanism is presented by the scheme in Figure 9. As the photon with energy associated with the same energy or larger than 3.16 eV or the light wavelength the same or smaller than 400 nm, there will be an electron excitation from the valence band into the conductance band that leads to the producing of radicals. The band gap energy of the photocatalyst was positioned at 3.16 eV, which is higher than that of water oxidation [E^0^ (H_2_O/·OH) = 2.38 V], and leads to the production of reactive hydroxyl radicals (·OH) to further oxidize MV via intermediates and completely form CO_2_ and H_2_O [42]. The redox potential of the CB of Zn-Al LDHs (−1.23 eV) [42] is lower than the potential of superoxide anion radicals (O_2_**^·^**^−^) [E^0^ (O_2_/O_2_**^·^**^−^) = −0.33 V], which means O_2_^**·**^^−^ will be produced easily and provide to generate peroxyl (HO_2_·) radicals [E^0^ (O_2_/HO_2_·). The hydroxyl radicals accelerate the oxidation mechanism [42,43]. The production of radicals was accelerated by the addition of H_2_O_2_ as an oxidant in the system. Propagation steps will be increased as the radicals produced by the interaction photocatalyst surface and light react to the H_2_O_2_ and lead the homolytic cleavage [32,44].

### 3.3. Photocatalyst Reusability

**Figure 10:**
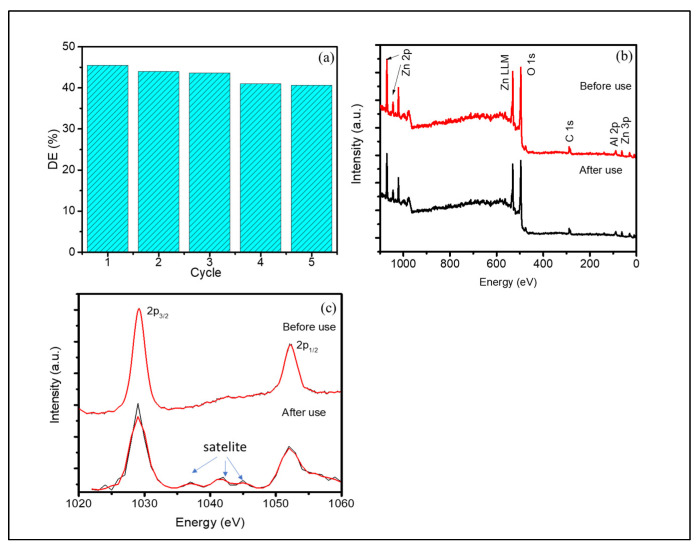
(**a**) Evaluation of DE at recyclability test using c-Zn-Al LDHs; (**b**) survey scan of sample before and after use; (**c**) spectra of Zn 2p before and after use.

Reusability is the important aspect for applicability in industrial-scale purposes. To check the reusability of photocatalyst, the recycling was conducted for the spent photocatalyst by filtering, washing with ethanol, and finally drying at 200 °C for 2 h. The recycled c-Zn-Al LDHs sample was further reused in the same treatment and the same condition. The DE of the recycled photocatalyst compared to the fresh one is presented in Figure 10a. The chart suggests that the DE remains stable within the range of 43–45% of the 5th cycle. This means that the photocatalyst is stable as shown by the reduced activity less than 10%. This stability performance was also confirmed by the XPS analysis presented in Figure 10b,c. The XPS spectrum shows the presence of Zn and Al as major components of the material without any significant change compared to the fresh sample. The survey scan spectrum (Figure 10b) shows the major peaks of Zn, O, and Al, clearly indicating the successful fabrication of c-Zn-Al LDHs, and the additional C peak is associated with the carbon tip used for analysis [18]. The intensity of the peaks of the after-use-sample remained unchanged, and there was no other atom identified. In the more-detailed analysis, there were some satellite peaks in the Zn 2p spectrum of the after-use-sample, which was attributed to the presence of other Zn species than Zn^2+^. This is usually associated with the change in some ZnO in the LDHs structure influencing the binding energy as the effect of oxidation reduction on the LDHs surface.

### 3.4. Photocatalytic Treatment of Peat Water

Similar with the photocatalytic oxidation of MV, the treatment was employed for the peat water sample. The parameter of the peat water utilized in this research is presented in Table 6.

**Figure 11:**
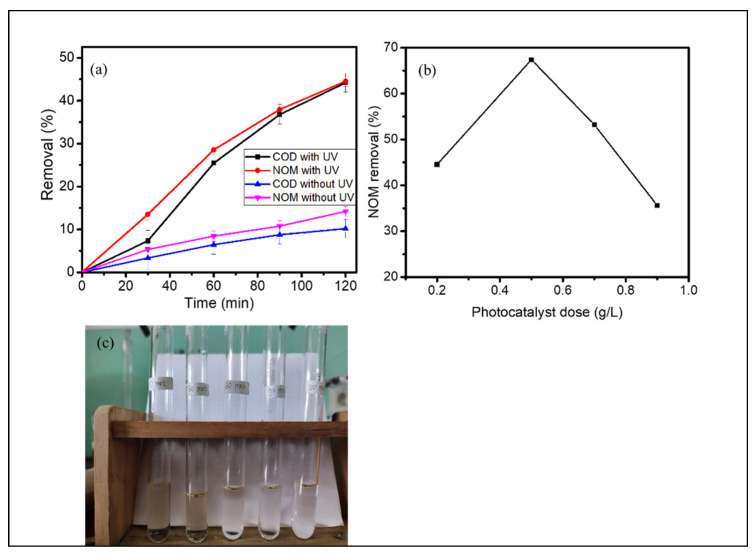
(**a**) Kinetics of NOM and COD removal of peat water photooxidation with and without UV light; (**b**) effect of c-Zn-Al LDHs photocatalyst dose on NOM removal by photooxidation with UV light; (**c**) some figures of initial and treated samples.

Kinetics of photocatalytic treatment on peat water using c-Zn-Al LDHs as a photocatalyst is depicted in Figure 11a and the effect of the photocatalyst dosage to the initial rate is presented in Figure 11b. The kinetics plots suggest that there is an effect of reducing NOM and COD by the photocatalytic oxidation with the maximum DE of about 44%. By comparing both UV_254_ and COD values that are not significantly different, it can be concluded that the organic matters in peat water is the main constituent causing the persistency of color and COD.

Furthermore, the effect of the photocatalyst dosage, in this case c-Zn-Al LDHs, as a photocatalyst shows an optimum condition of 0.5 g/L, which means that the higher photocatalyst dosage causes the ineffective oxidation process. Some figures from the experiment are presented in Figure 11c. From many studies on the effect of the photocatalyst amount on the photocatalytic reaction rate, the reduced activity at an increasing amount of photocatalyst is related with the limited light penetration for conducting the reduction–oxidation mechanism [32]. Moreover, the dark color of the peat water is the major problem in the whole process. Compared to the use of other photocatalysts such as TiO_2_ bead, the DE from this research is comparable [45,46]. Previous studies revealed that about 40% COD removal was achieved by using 250 mg/L TiO_2_ beads under UV illumination for 120 min. Referring to this study, the DE can be increased at an additional time of treatment [47].

## 4. Conclusions

The preparation of Zn-Al LDHs and the study of the effect of calcination on the photocatalytic activity of the material were conducted. The material characterization by using XRD, SEM, TEM, UV- Vis DRS, and XPS analyses confirmed the LDHs structure with the increasing interlayer distance, the specific surface area, and the band gap energy on the calcination procedure. The enhanced characters supported that the adsorption on the photocatalytic oxidation mechanism was by the adsorption isotherm studies as well as the studies on the effect of the initial concentration of MV and the effect of the photocatalyst dosage. Furthermore, reusability studies revealed the stability of material as shown by the insignificant change in DE and XPS analysis.

## Figures and Tables

**Table 1:** Calculated crystallite size from XRD measurement.

2θ	Zn-Al LDHs		c-Zn-Al LDHs	
	FWHM (unit)	Crystallite Size (nm)	FWHM (unit)	Crystallite Size (nm)
11.1	1.018	87.1	1.094	81.1
22.3	1.816	58.7	1.533	49.5
34.7	0.938	98.5	1.476	62.6
Crystallite size (nm)	81.4		64.4	

**Table 2:** Surface parameters from gas sorption analysis.

Surface Parameter	Zn-Al LDHs	c-Zn-Al LDHs
BET specific surface area (m^2^/g)	38.65	71.28
Pore volume (cc/g)	2.4 × 10^−2^	8.9 × 10^−2^
Pore radius (Å)	8.7	12.6

**Table 3:** Kinetics calculation of adsorption and photocatalytic oxidation.

	Process/Material	R^2^ of the Pseudo-First-Order Kinetics	R^2^ of the Pseudo-Second-Order Kinetics	Kinetics Constant (Pseudo-First-Order Kinetics-1/min)	DE at 120 min (%)
Adsorption	Zn-Al LDHs	0.968	0.956	1.04 × 10^−3^	17.54
	c-Zn-Al LDHs	0.994	0.991	1.72 × 10^−3^	18.53
Photooxidation	Zn-Al LDHs	0.997	0.905	2.96 × 10^−3^	41.28
	c-Zn-Al LDHs	0.998	0.984	3.37 × 10^−3^	45.57

**Table 4:** Calculated isotherm parameters.

Adsorbent	Freundlich Isotherm Parameters			Langmuir Isotherm Parameters			
	*K_F_* (L/g)	1/*n*	R^2^	*q_m_* (mg/g)	*K_L_* (L/mg)	R_L_	R^2^
Zn-Al LDHs	2.45	4.51	0.98	7.89	7.39 × 10^−3^	0.79	0.33
c-Zn-Al LDHs	2.64	4.46	0.97	9.02	9.03 ×10^−3^	0.98	0.55

**Table 5:** The comparison of MV degradation by photooxidation over various photocatalysts.

Photocatalyst	Remark	Light Wavelength/Source	Reference
ZnO nanoparticles	DE of 85% for 120 min photooxidation of MV 12.5 ppm by 1 g/L of photocatalyst	Sunlight	[38]
Zn-Cr LDHs	DE of 36% for 120 min photooxidation of 30 ppm MV by 1 g/L of photocatalyst	Solar simulator	[39]
PbTiO_3_	Photooxidation treatment for 120 min gave the DE of 90% on the initial MV concentration of 5 ppm and the photocatalyst dose of 3 g/30 mL	296 nm	[36]
ZnO nanorods	Photooxidation treatment for 120 min gave the DE of 90% for 10 ppm of MV and the dosage of 0.3 g/L	Sunlight	[34]
ZnO	Photooxidation treatment for 120 min gave the DE of 95% on the initial MV concentration of 10 ppm and the photocatalyst dose of 1.5 g/L	365 nm	[40]
ZnO	Photooxidation treatment for 120 min gave the DE of 96% on the initial MV concentration of 10 ppm and the photocatalyst dose of 1.5 g/L	Xenon lamp (Visible)	[41]
Zn-Al LDHs	Photooxidation treatment for 120 min gave the DE of 95.9% on the initial MV concentration of 10 ppm and the photocatalyst dose of 1.0 g/L	296 nm	This work

**Table 6:** Peat water parameters.

Parameter	Value
pH	6.2
TOC (mg/L)	1024
COD (mg/L)	655
UV254	2.87

## Data Availability

Not applicable.
